# Determinable and interpretable network representation for link prediction

**DOI:** 10.1038/s41598-022-21607-4

**Published:** 2022-10-20

**Authors:** Yue Deng

**Affiliations:** Institute of Fundamental and Frontier Sciences, University of Electronic Science and Technology of China, Chengdu, 611731 People’s Republic of China

**Keywords:** Complex networks, Information technology, Information theory and computation, Scientific data

## Abstract

As an intuitive description of complex physical, social, or brain systems, complex networks have fascinated scientists for decades. Recently, to abstract a network’s topological and dynamical attributes, network representation has been a prevalent technique, which can map a network or substructures (like nodes) into a low-dimensional vector space. Since its mainstream methods are mostly based on machine learning, a black box of an input-output data fitting mechanism, the learned vector’s dimension is indeterminable and the elements are not interpreted. Although massive efforts to cope with this issue have included, say, automated machine learning by computer scientists and learning theory by mathematicians, the root causes still remain unresolved. Consequently, enterprises need to spend enormous computing resources to work out a set of model hyperparameters that can bring good performance, and business personnel still finds difficulties in explaining the learned vector’s practical meaning. Given that, from a physical perspective, this article proposes two determinable and interpretable node representation methods. To evaluate their effectiveness and generalization, this article proposes Adaptive and Interpretable ProbS (AIProbS), a network-based model that can utilize node representations for link prediction. Experimental results showed that the AIProbS can reach state-of-the-art precision beyond baseline models on some small data whose distribution of training and test sets is usually not unified enough for machine learning methods to perform well. Besides, it can make a good trade-off with machine learning methods on precision, determinacy (or robustness), and interpretability. In practice, this work contributes to industrial companies without enough computing resources but who pursue good results based on small data during their early stage of development and who require high interpretability to better understand and carry out their business.

## Introduction

Physics has long been concerned as a propeller of civilization’s evolution in history. The establishment of Newtonian mechanics and thermodynamics drove the “first technological revolution”. The discovery of the electromagnetic induction phenomenon laid the theoretical foundation for the “second technological revolution”. Condensed matter physics and quantum physics developed the silicon semiconductor industry for the “third technological revolution”. With the ongoing “fourth technological revolution” currently, physics is also propelling the innovation and development in artificial intelligence, among which the study of complex networks^1,2^ is a case in point, since the input data of artificial intelligence models can be organized as networks, let alone that some artificial intelligence models have a network structure, like Graph Neural Network (GNN)^3^. By using nodes and edges to intuitively describe the nonlinear and heterogeneous interaction patterns of components composing the complex physical, social, and brain systems, the application of complex networks soon widened to various fields. For decades, scientists have been dedicated to understanding a network’s topological and dynamical attributes (like vital node identification^4,5^, high-order network topological analysis^6–9^, and percolation theory^10^) and to utilizing these attributes in specific applications, such as link prediction^11–15^, natural language processing^16^, and recommender systems^17,18^.

Recently, as a pivotal technique to abstract a network’s topological and dynamical attributes in a manner that maps the network or its substructures (like nodes) into a low-dimensional vector space, network representation^19,20^ has intrigued scientists for years, especially in light of ample evidence that network representation has several virtues dear to both academia and industry^18^, including reusable object representations by manual or automated feature engineering, enhanced model precision, and efficient parallel computation based on GPU. Nevertheless, since the mainstream methods of network representation are mostly based on machine learning^21^, almost a black box facing fundamental limits on well-explainable and raising difficulties in tedious hyperparameter tuning attributed to its input-output data fitting rationale, the dimension of the vector space to learn is indeterminable and the learned elements are not interpreted. Consequently, enormous computing resources are required for searching the sub-optimal dimension of the vector space within a range, and in most cases, researchers still can not interpret why such a dimension works out and what practical meanings the learned elements represent. Although recent years have seen massive efforts by computer scientists and mathematicians to cope with this issue, the root causes still remain unresolved. For example, although automated machine learning^22^ by computer scientists can pave the way for automating the search process of the space’s sub-optimal dimension, it still requires enormous computing resources. Moreover, although mathematicians can conclude some empirical formulas used to calculate the possible optimal dimension for the space^23^, each of them is normally with respect to specific models and only can be taken as statistical results from specific data, limiting their generalization to other scenarios. Given these inadequacies, determinable and interpretable network representation is still an open and important question.

Differently, from a physical perspective, this article proposes two methods of determinable and interpretable network representation. The first method is based on the Degree, H-index, and Coreness (DHC) theorem^24^ that constructs an operator to generate H-indices sequences (with a determined length) for nodes. Regarding their practical meanings and on the advice of the rich club theory^25^, this article utilizes these H-indices sequences to construct node representations, which can be used to abstract nodes’ local topological and dynamical attributes related to their neighborhood. To abstract nodes’ global attributes related to the whole network, the second method is based on the DHC Entropy (DHC-E)^26^, a hyperparameter-free and explainable whole graph embedding algorithm we proposed. If a bipartite network containing $m+n$ nodes of two types, its corresponding $m \times n$ adjacent matrix can be extended to a $(m+n) \times (m+n)$ augmented matrix, a simple matrix that can be decomposed to $m+n$ matrices, each of which corresponds to a node and carries the node’s global attributes. After implementing the DHC-E algorithm on each of them, node representations can be generated. Unlike those learned by machine learning-based methods, the node representations generated by the two methods have both a determined dimension and interpretable elements.

To evaluate the two methods’ effectiveness and generalization, this article further proposes Adaptive and Interpretable ProbS (AIProbS), a network-based link prediction model for bipartite networks, which can utilize nodal representations to measure the similarity between nodes. Methodologically, built on a classical network-based framework called Probabilistic Spreading (ProbS)^27^, the AIProbS can control the resource diffusion process of the ProbS framework by setting edge weights quantified with the similarity between nodes measured by node representations. By doing so, the AIProbS makes the flaw of the classical ProbS framework in self-adaptive perception (or pattern recognition) ability oriented to different scenarios (i.e., different complex networks), since the node representations involved can perceive, abstract, and carry underlying patterns of different complex networks, which is inspired from the feature recognition function of machine learning methods. At the same time, compared with machine learning-based link prediction models^28–32^, the AIProbS is hyperparameter-free, meaning that its implementation process is determinable and the results are interpreted. In addition, implemented on several designed control experiments of diverse recommender systems (a specific application of link prediction in artificial intelligence), experimental results showed that the AIProbS can reach state-of-the-art precision beyond baseline models on some scenarios with small data and can, by and large, make a good trade-off with machine learning-based models on precision, determinacy, and interpretability.

## Methods

In the first place, this article proposes two novel network representation methods, which are determinable and interpretable. Then, a classical network-based link prediction framework called Probabilistic Spreading (ProbS) is introduced, and its flaws are revealed. Based on the ProbS framework, this article proposes Adaptive and Interpretable ProbS (AIProbS), a network-based link prediction model for bipartite networks, which can utilize nodal representations generated by the two methods and can enhance the precision of link prediction beyond the classical ProbS framework after making up its flaws.

### Generate a complex network’s nodal representations

#### Method one

A complex network is comprised of nodes and edges, exhibiting various macroscopic dynamic attributes determined by its different microscopic structures (i.e., different connection patterns between nodes). To quantify nodal influence, one well-studied topological characteristic of a complex network *G*, degree, H-index, and coreness are three common-used measurements:

##### Definition 2.1

 For any node $v_i \in G$, if there are $k_i$ neighbors of $v_i$ (i.e. $k_i$ nodes connected with $v_i$), the degree of $v_i$ is $k_i$.

##### Definition 2.2

 For any node $v_i \in G$, if nodes $v_{j_1}, v_{j_2}, ..., v_{j_{k_i}}$ with degrees $k_{j_1}, k_{j_2}, ..., k_{j_{k_i}}$, respectively, are the $k_i$ neighbors of $v_i$, the H-index^33^ of node $v_i$ is the maximum value *h* such that among these $k_i$ neighbors, it has at least *h* neighbors with a degree no less than *h*.

##### Definition 2.3

For any node $v_i \in G$, its coreness is calculated by *k*-core decomposition^34^ (see Fig. S1 in Appendix D for an example).

Intuitively, the greater a node’s degree is, the more neighbors it is connected with, and the higher influence it has, and so does a node’s H-index. Furthermore, to take location into account, a node’s coreness can be used to reveal its centrality: a greater coreness indicates that a node locates more centrally in a complex network and hence has a higher influence.

The DHC theorem^24^ reveals that degree, H-index, and coreness are all related, that is, the coreness of nodes in a network can be derived from their degree values. To mathematically describe the relationship, the DHC theorem constructs an operator ${\mathcal {H}}$, which calculates the maximum value *h* for each node such that the node has at least *h* neighbors with H-indices no less than *h*. For each node *i* in a complex network, taking its degree $k_i$ as the zero-order H-index $h_i^{(0)}$ as the beginning, the first-order H-index $h_{i}^{(1)}$ of node *i* is calculated by ${\mathcal {H}}(h_{j_1}^{(0)}, h_{j_2}^{(0)}, ..., h_{j_{k_i}}^{(0)})$, where $h_{j_1}^{(0)}, h_{j_2}^{(0)}, ..., h_{j_{k_i}}^{(0)}$ are the zero-order H-indices (i.e., the degree values) of the $k_i$ neighbors of node *i*. By iteratively doing so, $h_i^{(2)} = {\mathcal {H}}(h_{j_1}^{(1)}, h_{j_2}^{(1)}, ..., h_{j_{k_i}}^{(1)})$, as well as $h_i^{(3)}, h_i^{(4)},...$, can be calculated. Finally, a sequence $h_i^{(0)}, h_i^{(1)}, h_i^{(2)}, ...$ with a fixed length (because the sequence is finally convergent to the node’s coreness) is generated for node *i*, as the DHC theorem states:

##### Theorem 2.1

*For each node in a complex network, node* *i’s H-indices sequence* $h_i^{(0)}, h_i^{(1)}, h_i^{(2)}, ...$ *is convergent to its coreness* $c_i$, *i.e.*, $\displaystyle c_i = \lim _{n \rightarrow \infty } h_i^{(n)}$.

##### Proof

See^24^. $$\square $$

##### Definition 2.4

Given a complex network *G* with *n* nodes. If there exists a mapping $\Phi : G \rightarrow {\mathbb {R}}^{n \times k}$ that can project *G* into a Euclidean space ${\mathbb {R}}^{n \times k}$, where *k* ($k \ll n$) is the Euclidean space’s dimension, then vector $\Phi (v_i) \in {\mathbb {R}}^k$ is taken as the nodal representation of node *i*.

Effective nodal representations are supposed to fully perceive and preserve a complex network’s topological characteristics and can be used to approximately reconstruct the network. Conforming to the definition of nodal representations, the H-indices sequences generated by the DHC theorem can be taken as effective nodal representations for the following reasons.

First, according to the rich club theory^25^ (from the field of social network analysis^35^ and soon widened to interdisciplinary studies like computer science^36^ and cognitive science^37^) that a node’s influence could reflect its role in its neighborhood or the whole network, this article proposes the following assumption:

##### Assumption 2.1

*A node’s H-indices sequence can abstract the node’s multidimensional influence in the neighborhood, where the sequence’s convergence steps can reflect the magnitude of the node’s influence. The more important role played by the node in the neighborhood, the more slowly its influence decays during the dynamic evolution (i.e., the convergence process by the DHC theorem), thus the larger its convergence steps are.*

Then, built on Assumption 2.1 this article takes a node’s H-indices sequence as its nodal representation. In this way, provided *n* nodes in a complex network and given that their H-indices sequence converges after up to *s* steps, this method can not only mine out and carry a network’s underlying patterns but also map the *n* nodes to a *s*-dimensional vector space consisting of their H-indices as nodal representations, both of which simulate the key target of machine learning methods. Differently, this is a determinable and interpretable network representation method, since for an arbitrary complex network the dimension of its nodal representations is determined as *s* and the elements can be interpreted as nodal multidimensional influence with different magnitudes.

Conceivably, one must stress why the DHC theorem is chosen but not others. One motivation lies in that although the field of complex networks has sprouted a range of findings that can represent nodal multidimensional influence, like directly concatenating several metrics used to measure nodal influence together as features with a fixed dimension, the DHC theorem seems to be a more appropriate one that can generate something being taken as nodal representations with an adaptive dimension, conforming to definition 2.4, and that can simulate the essential target of machine learning in pattern recognition. This is the first work assuming that the DHC theorem plays a role in representing nodal multidimensional influence.

#### Method two

Following method one, to further abstract a node’s global attributes in a complex network, if a bipartite network with $m+n$ nodes of two types (see Appendix D for its definitions and illustrations), its adjacency matrix $A^{m \times n}$ can be extended to $B^{(m+n)\times (m+n)}$ constructed by $$\displaystyle \left( \begin{array}{cc} O^{m \times m} &{} A^{m \times n} \\ (A^{m \times n})^T &{} O^{n \times n} \end{array}\right) $$, where *O* denotes the null matrix. Based on it, a series of $\lambda _i$ and $B_i$ can be decomposed by the following theorem.

##### Theorem 2.2

*The adjacency matrix* $\displaystyle B^{(m+n)\times (m+n)}$ *can be decomposed by* $\displaystyle B=\sum _{i=1}^{m+n}\lambda _i B_i$, *where* $\lambda _i$ *is the i-th eigenvalue of* $\displaystyle B^{(m+n)\times (m+n)}$ *and* $\displaystyle B_i$ *is the corresponding idempotent matrix.*

##### Proof

See Appendix A. $$\square $$

After that, this article implements the DHC-E operator ${\mathcal {E}}$^26^ (i.e., resort the DHC theorem as a strategy for generating a H-index matrix $H^{n \times s}$ by row containing the H-indices converged after *s* steps of each of the *n* nodes in a complex network, the operator ${\mathcal {E}}$ calculates the Shannon entropy of each column of $H^{n \times s}$ and obtains a vector $e^{1 \times s}$, as the whole graph embedding of the network) on each $B_i$ or $\lambda _i B_i$ one by one, generating the $m+n$ nodes’ representations for the bipartite network correspondingly. Apparently, this method is also a determinable and interpretable network representation method. The characteristics of interpretability and hyperparameter-free of the DHC-E algorithm are thoroughly illuminated in our previous works^26^. See Appendix D for intuitive illustrations on the two methods.

### The ProbS framework and its flaws

To evaluate the two methods’ effectiveness and generalization, this article utilizes them in link prediction for bipartite networks. Since network representation can be used to perceive and abstract a complex network’s underlying topological and dynamical attributes, the key target (i.e., pattern recognition) of machine learning, this article explores how nodal representations generated by the two methods can be utilized in link prediction, aiming to enhance the precision of conventional (non-machine learning-based) prediction models.

Among conventional link prediction models for bipartite networks, the Probabilistic Spreading (ProbS)^27^ framework has been recognized as a typical one. By means of a resource diffusion mechanism inspired by the physical process of Material Diffusion, the ProbS framework can quantify the similarity between nodes after initializing and diffusing resources. Fig. S1 in Appendix D includes an example to intuitively illuminate the schematics of the ProbS framework. For instance, in the bipartite network with nodes of two types (i.e., nodes *A*, *B*, and *C* are of type one, and nodes *a*, *b*, *c*, and *d* are of type two), when predicting node *B*’s unobserved links with nodes *a* and *b*, resources are first initialized at nodes *c* and *d* (the nodes that are connected with node *B*) with value 1, then are diffused to nodes *A*, *B*, and *C* along edges after being equally divided by the degree of each node, finally are diffused back to nodes *a*, *b*, *c*, and *d* in the same way, which can be used to quantify the similarity between node *B* and the four nodes, respectively. A larger similarity of two nodes indicates a higher probability of an unobserved link existing between them.

This article provides a mathematical perspective to describe the ProbS framework, by constructing an operator *T* to describe its diffusion mechanism. Given a bipartite network consisting of $m+n$ nodes of two different types, respectively, whose adjacency matrix is represented by $A^{m \times n}$. Let $R^{m \times n}$ denote the predicted matrix, where $R_{ij}$ represents the similarity (i.e., the probability of the existence of a link) between nodes *i* and *j*. Then, through the ProbS framework $R^{m \times n}$ can be calculated by

1 $$R = A \cdot (D_{I} \circ A)^T \cdot (D_{U} \circ A)$$

where $\cdot$ denotes the dot product, and $\circ$ denotes the Hadamard product. $D_I^{m \times n} = (a_1, a_2, ..., a_n)$, $\displaystyle a_i = (\frac{1}{k_{I_i}}, ... , \frac{1}{k_{I_i}})^T$ where $k_{I_i}$ is item *i*’s degree. $D_U^{m \times n} = (a_1, a_2, ..., a_m)^T$, $\displaystyle a_i = (\frac{1}{k_{U_i}}, ... ,\frac{1}{k_{U_i}})$ where $k_{U_i}$ is user *i*’s degree. In Eq. (1) the operator $T=(D_{I} \circ A)^T \cdot (D_{U} \circ A)$.

This algebraic form of the ProbS framework can directly be used to support parallel computing. In addition, the operator *T* illuminates why the diffusion process of ProbS framework converges in a manner that deriving *R* from *A* and then placing *A* with the derived *R* iteratively, stated as the following theorem.

#### Theorem 2.3

Let the operator $T=(D_{I} \circ A)^T \cdot (D_{U} \circ A)$ iteratively act on *A* by $A \leftarrow A \cdot T$, the iterative process is convergent.

#### Proof

See Appendix B. $$\square $$

Since the difference between the values in *A* tends to be smoother as the convergent iterative process progresses while link prediction relies for higher precision on the more distinctive differentiation between the predicted values of similarity^18^, in link prediction the best iteration steps for the ProbS framework is 1.

Most importantly, from such a mathematical perspective, it is intuitive to see that the ProbS framework faces fundamental limits on self-adaptive perception (or pattern recognition) ability because its resource diffusion mechanism is just based on equal allocation, shown as $D_I$ and $D_U$ in Eq. (1). In practice like recommender systems (an application of link prediction for bipartite networks in artificial intelligence), such a mechanism raises a key question: if respectively take these nodes of two different types as users and items in recommender systems, the resources diffused between users and items back and forth, to some extent, represent user’s preferences for items or item’s attractiveness to users, while neither of them should be necessarily equal since user biases^38–40^ and item biases^38,41^ generally exist in reality. Moreover, these biases are usually recommendation scenario-oriented, which means that in different scenarios a user’s preferences may differ, and so do an item’s attractiveness or popularity. Finally, in practice the ProbS framework fails to take these biases into consideration, let alone adaptively perceive and quantify their differences in various scenarios.

### The AIProbS model

The essential condition for the ProbS framework to make up for its flaws is to be equipped with self-adaptive perception (or pattern recognition) ability, in attempting to abstract and utilize the attributes of nodes (represented by nodal representations) in complex networks toward different scenarios. To utilize the nodal representations generated by the two proposed methods in the ProbS framework, this article proposes Adaptive and Interpretable ProbS (AIProbS).

In the first step, on the advice that the rich club theory^25^ gives clues that nodes with high centrality tend to form tightly interconnected communities, this article generalizes this conclusion to the field of link prediction, proposing the following assumption:

#### Assumption 2.2

*The similarity between node pairs having strongly correlated nodal representations (i.e., similar features or similar influence) is higher than that between weakly correlated ones*.

To measure the similarity between nodes, the AIProbS uses the cosine similarity metric. Provided two *n*-dimension vectors *x* and *y*, the cosine similarity between them is calculated by $\displaystyle \cos (\theta )=\frac{x \cdot y}{|x| \cdot |y|}=\frac{\sum _{i=1}^n x_i y_i}{\sqrt{\sum _{i=1}^n x_i^2} \cdot \sqrt{\sum _{i=1}^n y_i^2}}$. In the same way, provided $m+n$ nodes belonging to two sets *U* and *I* of two different types in a bipartite network, respectively. Through the network representation methods proposed in this article the representation matrices $F_U^{m \times s}$ and $F_I^{n \times s}$ of the two types of nodes are generated, either of which is consist of nodal representations by row. Then, the $m \times n$ nodal similarity matrix $S^{m \times n}$ calculated by the cosine similarity metric is

2 $$\begin{aligned} S = \frac{F_{U} \cdot F_{I}^T}{\alpha ^T \cdot \beta }, \end{aligned}$$

where vector $\displaystyle \alpha = \big (\sqrt{\sum _{j=1}^s{F_U}_{1j}^2}\,,\sqrt{\sum _{j=1}^s{F_U}_{2j}^2}\,, \,...\,, \sqrt{\sum _{j=1}^s{F_U}_{mj}^2}\big )$ and vector $\displaystyle \beta = \big (\sqrt{\sum _{j=1}^s{F_I}_{1j}^2}\,,\sqrt{\sum _{j=1}^s{F_I}_{2j}^2}\,, \,...\,, \sqrt{\sum _{j=1}^s{F_I}_{nj}^2}\big )$.

After obtaining the nodal similarity matrix $S^{m \times n}$, utilizing it to control the diffusion process of the classical ProbS framework is the second step. To assign proper weights to every node pair for the diffusion mechanism of the ProbS framework, the AIProbS further complete some normalization and proportioning operations on $S \circ A$ where *A* is the adjacency matrix shown in Eq. (1). Since the elements of $S \circ A$ vary in $[-1,1]$ while the diffused resources are supposed to be positive, the AIProbS normalizes the value range of the elements to [0, 1] using the max–min normalization operation, for each row vector $(S\circ A)_{i*}$ $(i=1,2,...,m)$ of $S \circ A$, by

3 $$\begin{aligned} (S \circ A)_{ij} \leftarrow \frac{(S \circ A)_{ij} - \min }{ \max - \min },\quad \,j = 1,2, ..., n, \end{aligned}$$

where the $\max$ and $\min$ are the maximum and minimum elements of the row vector $(S \circ A)_{i*}$, respectively. Based on that, the weight matrix $W_U$ for nodes belonging to set *U* is calculated by the proportioning operation as

4 $$\begin{aligned} W_{U_{ij}} = \frac{1}{(S \circ A)_{ij}}\sum _{k=1}^{n} (S \circ A)_{ik}, \,i=1,2,...,m, \quad \, j=1,2,...,n. \end{aligned}$$

On the other hand, the same operations are completed on $S^{m \times n}$ by column, generating the weight matrix $W_I^{m \times n}$ for nodes belonging to set *I*.

In the last step, the predicted matrix $R^{m \times n}$, where $R_{ij}$ represents the prediced similarity between nodes *i* and *j*, is calculated through the AIProbS by

5 $$\begin{aligned} R = A \cdot W_{I}^T \cdot W_{U}. \end{aligned}$$

Conceivably, there are other metrics for similarity measurement. More combinations were tested in this article (see Appendix C for details) but none of them performed better than the one used in this section. All in all, the whole process of the AIProbS are summarized in the pseudocodes shown in Appendix D. For more intuitive illumination, Fig. S1 in Appendix D presents the schematics of the AIProbS.

### Performance evaluation

To evaluate the precision of the AIProbS as well as its pros and cons in link prediction for bipartite networks, from which the effectiveness of nodal representations generated by the two proposed nodal representation methods can be reflected, this section designs control experiments based on recommender systems, an application of link prediction in artificial intelligence.

#### Recommender systems

By analyzing observed user-item relations (see Appendix D for illustrations) to predict a user’s preferred items from millions of candidates, recommender systems^17,18,42–44^ are recognized as a pivotal tool to alleviate the information overload problem. Among different user-item relations, implicit user-item interactions (e.g., user’s historical clicks or buys on items) record the existence of a user’s interactions with items, defined as a binary state using 1 and 0. From the perspective of a complex network, recommendation on implicit user-item interactions can be seen as a process of link prediction for bipartite networks, where users and items correspond to the two types of nodes and implicit user-item interactions represent the edges between nodes. Therefore, the experiments designed in this section are built on recommender systems with implicit user-item interactions, for most recommendation models are based on them.

#### Data sets

In light of the no-free-lunch theorem^45^ that no model can always perform well as expected in different scenarios, this section designs control experiments to evaluate the performance of the AIProbS on diverse real recommendation scenarios, in order to explore not only the pros of the AIPobS but also its cons in different scenarios.

**Table 1:** Overview of data sets.

Data sets	|*U*|	|*I*|	Interactions	Sparsity (%)
MovieLens 100K	943	1680	100,000	93.70
MovieLens 1M	6040	3952	1,000,209	95.81
LastFM	1892	17,632	92,834	99.72

As shown in Table 1, |*U*| and |*I*| represent the number of users and items, respectively, and the interactions between users and items are implicit ones. The sparsity in Table 1 represents the ratio of the number of unobserved interactions to the maximum number of all possible interactions between users and items (e.g., that between *m* users and *n* items is *mn*). As a control group, the MovieLens 100K, MovieLens 1M, and LastFM are three classical data sets from two different recommender systems of movies and music, with distinctive ratios of |*U*| to |*V*|, data scales, and sparsity, based on which more persuadable results could yield compared with those based on newly published data sets, since these classical data sets have been widely used for evaluation in previous works.

In order to guarantee the reproducibility of experiments, either of the three data sets is obtained from the RecBole public resources (https://recbole.io/dataset_list.html), organized into tuples (user, item, 0/1) without preprocessing. Each of them is randomly split into a “train/evaluate/test” set by the ratio of “$80/10/10\%$”. After independently repeating the splitting process 30 times, 30 realizations are generated for each data set.

When speaking of the scales of data sets, one is tempted to say that the used ones in this article are not big. Indeed, in the popular conception, the bigger the data set is, the better precision a machine learning-based model can reach, spring from its data fitting mechanism. Nevertheless, in practice, not every enterprise could afford the implementation of a machine learning-based model, which are highly computing-resource consuming for hyperparameter tuning. In addition, in the early stage of a company’s development, there would be not enough data supporting a well-fit machine learning-based model. Recognizing that, this work aims to make up for this situation and therefore mainly concentrates on the implementation of models on data sets with small and middle scales. In fact, it has been rare to see a machine learning model implemented on small data sets. So, it would be interesting and valuable to make up for this blank.

#### Evaluation metrics

In order to quantify the precision of the AIProbS on these data sets, three common-used metrics are chosen in this article. Given a user $u \in U$ (*U* is the user set) and the length *N* of the recommendation list, the set of recommended items for the user is denoted by ${\hat{R}}(u)$ and the ground-truth set of items the user interacted with is denoted by *R*(*u*). Based on them, the first evaluation metric is the Recall@N^46^, which calculates the fraction of predicted relevant items out of all ground-truth relevant items by

6 $$\begin{aligned} \text{ Recall@N }=\frac{1}{|U|}\sum _{u \in U} \frac{|{\hat{R}}(u) \cap R(u)|}{|R(u)|}, \end{aligned}$$

where |*R*(*u*)| represents the item count of *R*(*u*).

To calculate the reciprocal rank of the first relevant item recommended to each user, the second evaluation metric Mean Reciprocal Rank (MRR@N)^47^ is denoted as

7 $$\begin{aligned} \text{ MRR@N }=\frac{1}{|U|} \sum _{u \in U} \frac{1}{\text{ rank}_u^*}, \end{aligned}$$

where $\text{ rank}_u^*$ is the rank position of the first relevant item recommended to user *u*.

Moreover, as the third evaluation metric, the Normalized Discounted Cumulative Gain (NDCG@N)^48^ can further measure the overall ranking quality in a manner that accounts for the position of the hit by assigning higher scores to hits at top ranks as

8 $$\begin{aligned} \text{ NDCG@N }=\frac{1}{|U|} \sum _{u \in U} \big ( \frac{1}{\sum _{i=1}^{\min (|R(u)|,N)}\frac{1}{\log _2(i+1)}} \sum _{i=1}^N \delta (i \in R(u)) \frac{1}{\log _2(i+1)}\big ), \end{aligned}$$

where $\delta (\cdot )$ is an indicator function and positions are discounted logarithmically.

In practice, the greater the values of these evaluation metrics are, the higher a model’s precision is.

#### Baseline methods

This article chooses nine baseline models as follows, evaluating the pros and cons of the AIProbS compared with its predecessors of both conventional and machine learning-based ones.

Conventional baselines include two models. As the bedrock, the Probabilistic Spreading (ProbS)^27^ is a necessary baseline used to evaluate the improvement of the AIProbS. In addition, one might expect to base the recommendation directly on the nodal representations generated by the two proposed methods, not built on the ProbS framework. To test this strategy, this section constructs the Pure-DHC model, used to perform the recommendation by Eq. (2) based on the user-item similarity of their H-indices (i.e., nodal representations). One might also expect that why not directly use similarity metrics like Jaccord and Adamic-Adar in link prediction without through the ProbS framework? Previous works have proved that the ProbS framework outperformed those directly used metrics for link prediction^17,27^. In view of it, to evaluate its improvement beyond other conventional methods, the AIProbS is only needed to be compared with the ProbS framework.

Machine learning-based baselines include seven models. To avoid the baseline pitfalls that have plagued earlier research on the comprehensive and objective evaluation of proposed models, this article further chooses eight representative machine learning-based models as baselines, among which were based on six different techniques of machine learning frameworks, including Neural Matrix Factorization (NeuMF)^49^ based on deep neural networks, Convolutional Neural Collaborative Filtering (ConvNCF)^50^ and Spectral Collaborative Filtering (SpectralCF)^51^ based on convolution operations, Graph Convolutional Matrix Completion (GCMC)^52^ based on graph auto-encoder frameworks, LINE^53^ based on random walking, Neural Graph Collaborative Filtering (NGCF)^54^ based on graph neural networks, and Disentangled Graph Collaborative Filtering (DGCF)^55^ and Neighborhood-enriched Contrastive Learning (NCL)^56^ based on attention mechanisms.

## Results

Based on the experimental settings, this section presents the experimental results on the precision, robustness, and interpretability of the AIProbS and baseline models, revealing their pros and cons in different recommendation scenarios.

### Precision analysis

As shown in Tables 2, 3 and 4, the results on model precision are presented, where the length *N* of the recommendation list is set to 10, and each model’s precision is averaged from its independently implementation based on 30 different realizations. The values in parentheses indicate the percentage of improvement or decline in model precision of the AIProb model compared to each of the baseline models based on each data set and evaluated by each metric, where the percentage of improvement is bold. The best and the second-best results for each column are highlighted by italics and bold italics fonts.

Conceivably, when speaking of the necessity of determinable and interpretable network representation and their utilization in link prediction, one might cast it into doubt: why not base link prediction directly on the nodal representations generated by the two proposed methods (as the Pure-DHC model does) but built on the AIProbS? Can the precision of the conventional ProbS framework really be enhanced by being involved with nodal representations as an ability in pattern recognition? Why do we need the AIProbS when machine learning-based methods have long been recognized to be more precise on big data? These questions are answered point to point in the rest of this section. As shown in Tables 2, 3 and 4, on all three data sets the Pure-DHC which directly utilizes the nodal representations generated by the proposed methods in recommendation achieved the worst prediction precision among the AIProbS and baseline models. That is to say, such generated nodal representations could be nothing with the recommendation if not utilized in the ProbS framework. After utilizing these nodal representations in the ProbS framework, as shown in Tables 2, 3, and 4, the AIProbS outperformed the conventional ProbS on all three data sets. However, when compared to machine learning-based baselines, the AIProbS indeed performed worse than some of them, most obviously on MovieLens 1M. But it still can achieve state-of-the-art performance on prediction precision on LastFM, suggesting that nodal representations generated by the proposed methods may be able to perceive and abstract the underlying patterns hidden in a complex network, which can be used to enhance the performance of recommendation methods.

**Table 2:** Results of model precision on LastFM.

Models	LastFM		
	Recall@10	MRR@10	NDCG@10
Pure-DHC	0.004	0.006	0.003
ProbS	0.170 (**+8.2**%)	0.308 (**+10.4**%)	0.166 (**+10.2**%)
AIProbS	*0.184*	*0.340*	*0.183*
NeuMF	0.060 (**+206.7**%)	0.092 (**+269.9**%)	0.050 (**+266.0**%)
ConvNCF	0.056 (**+228.6**%)	0.090 (**+277.8**%)	0.048 (**+281.3**%)
SpectralCF	0.066 (**+178.8**%)	0.120 (**+183.3**%)	0.062 (**+195.2**%)
GCMC	0.121 (**+52.1**%)	0.214 (**+58.9**%)	0.116 (**+57.8**%)
LINE	0.149 (**+23.5**%)	0.272 (**+25.0**%)	0.145 (**+26.2**%)
NGCF	0.169 (**+8.9**%)	0.301 (**+13.0**%)	0.163 (**+12.3**%)
DGCF	0.177 (**+4.0**%)	0.316 (**+7.6**%)	0.172 (**+6.4**%)
NCL	***0.183*** (***+0.3%***)	***0.337*** (***+0.8%***)	***0.182*** (***+0.6%***)

The best and the second-best results for each column are highlighted by italics and bold italics fonts.

**Table 3:** Results of model precision on MovieLens 100K.

Models	MovieLens 100K		
	Recall@10	MRR@10	NDCG@10
Pure-DHC	0.017	0.084	0.041
ProbS	0.208 (**+3.4**%)	0.413 (**+5.1**%)	0.236 (**+5.1**%)
AIProbS	**0.215**	**0.434**	**0.248**
NeuMF	0.070 (**+207.1**%)	0.187 (**+132.1**%)	0.093 (**+166.7**%)
ConvNCF	0.099 (**+117.2**%)	0.245 (**+77.1**%)	0.125 (**+98.4**%)
SpectralCF	0.124 (**+73.4**%)	0.293 (**+48.1**%)	0.153 (**+62.1**%)
GCMC	0.196 (**+9.7**%)	0.400 (**+8.5**%)	0.232 (**+6.9**%)
LINE	0.190 (**+13.2**%)	0.391 (**+11.0**%)	0.225 (**+10.2**%)
NGCF	*0.245* (−*12.2*%)	*0.481* (−*9.8*%)	*0.293* (*−15.4%*)
DGCF	0.236 (−8.9%)	0.458 (−5.2%)	0.278 (−10.8%)
NCL	***0.240 ***(*−10.4% *)	***0.469 ***(*−7.5% *)	***0.285 ***(*− 13.0%*)

The best and the second-best results for each column are highlighted by italics and bold italics fonts.

**Table 4:** Results of model precision on MovieLens 1M.

Models	MovieLens 1M		
	Recall@10	MRR@10	NDCG@10
Pure-DHC	0.002	0.031	0.014
ProbS	0.108 (**+21.3%**)	0.352 (**+17.6%**)	0.177 (**+18.6%**)
AIProbS	**0.131**	**0.414**	**0.210**
NeuMF	0.032 (**+309.4%**)	0.128 (**+223.4%**)	0.053 (**+296.2%**)
ConvNCF	0.073 (**+79.5%**)	0.255 (**+62.4%**)	0.128 (**+64.1%**)
SpectralCF	0.147 (−10.9%)	0.416 (−0.5%)	0.236 (−11.0%)
GCMC	0.152 (−13.8%)	0.421 (−1.7%)	0.240 (−12.5%)
LINE	0.153 (−14.4%)	0.423 (−2.1%)	0.236 (−11.0%)
NGCF	0.162 (−19.1%)	0.442 (−6.3%)	0.254 (−17.3%)
DGCF	***0.172*** (− *23.8%*)	***0.460*** (− *10.0%*)	***0.266*** (− *21.1%*)
NCL	*0.180* (− *2*7.2%)	*0.482* (− *14.1*%)	*0.279* (− *24.7*%)

The best and the second-best results for each column are highlighted by italics and bold italics fonts.

To put these results in more general terms, it is definite that designing control experiments to guarantee the comprehensiveness and objectivity of model performance evaluation is indispensable because, as shown in Tables 2, 3 and 4, the comparative predominance between different models or even that between the conventional and machine learning-based models are distinctive. For instance, compared to its predecessor (the ProbS framework), the AIProbS at best improved the Recall@10 by $21.3\%$ on MovieLens 1M and at worst, by $3.4\%$ on MovieLens 100K. Such a $17.9\%$ gap shows that the predominance of the AIProbS over the ProbS is not necessarily that significant in all recommendation scenarios. Overall, on MovieLens 1M, although it achieved an appreciable improvement over the ProbS, the AIProbS still performed worse than the other six machine learning-based models, revealing the predominance of the machine learning-based frameworks over the conventional ones on this data set. Nonetheless, that predominance faded on MovieLens 100K because only three machine learning-based models (i.e., NGCF, DGCF, and NCL) outperformed the AIProbS. On LastFM, none of the machine learning-based models outperformed the AIProbS, in other words, but the AIProbS achieved state-of-the-art performance on prediction precision.

Figuring out the determinant factors of model performance in different recommendation scenarios is not easy and intuitive, not to mention accurately predicting a model’s performance for one specific scenario. Still, on the advice of the clues given in Tables 2, 3 and 4, some discoveries could be summarized as follows. (1) The machine learning-based models might have a predominance on data sets with large scales. The recommendation scenario of MovieLens 1M and MovieLens 100K being equal, the machine learning-based models showed a more distinguished predominance on the former with a comparatively larger data scale than the latter. However, it is hard to assert that the distinctions of ratios of |*U*| to |*V*| and the sparsity of the two data sets play a silent role. (2) The conventional ProbS framework might play a large role in recommendation scenarios with high sparsity. Since the sparsity of LastFM is the highest among the three data sets, where the machine learning-based models face fundamental limits on lack of enough user-item interactions for training, the AIProbS or the ProbS combined with or of the conventional frameworks showed their predominance as a result of their network structure-oriented resolution. Nevertheless, the ratio of |*U*| to |*V*| of LastFM, which seems to be a little higher than the other two, could also be a decisive factor.

As shown in Tables 2, 3, and 4, one might suspect the precision of NeuMF, ConvNCF, and SpectralCF, which can perform well on some big data but are seemingly aberrant here. Since one of this article’s focuses is to produce substantial benefits for industrial companies without enough computing and data resources, even though the three models may work better when being set with more layers and larger dimensions in their model structures and higher early stop steps for the training, it is still worth exposing their flaws in this article’s specific scenarios by implementing them based on a resource-consuming condition (meaning that their hyperparameters were not set very large and the early stops were not set far more than other baselines’ that requires for a long-running time for the three models’ convergence). In other words, their poor performance in some realizations under this constrained condition was not eliminated but averaged into the precision results. In view that there should be more than precision to analyze, the following sections further discuss the robustness and interpretability of the AIProbS and baselines.

### Robustness analysis

As revealed in the previous section, with the increase in data scale the precision of the AIProbS decreased compared with that of machine learning-based baselines, for the mechanism of data fitting (or pattern recognition) adopted by machine learning methods can give fully to its play more suitably in scenarios with larger data scale. Nevertheless, it does not mean that the AIProbS is superfluous in those scenarios. Since tedious hyperparameter tuning is required for up to the optimal performance of a model, the model’s computing efficiency reduction (containing the preparation time paid for hyperparameter tuning) is inevitable for machine learning methods to reach their expected precision. Such a strategy brings about heavy financial (i.e., computing resources) and time costs for the implementation of these methods built on big data. Even though at best, a well-trained machine learning-based model with identified hyperparameters can be acted on end tasks after fine-tuning, the original tuning process is still inevitable prior to that. At worst, if the model is applied to another scenario with different data distribution from that where it was trained, it has to be retrained before working, in order to identify a new group of hyperparameters with respect to the changed scenario. In contrast, the nodal representation generated by the proposed methods is determinable in dimension and elements, and can be directly used in the AIProbS, indicating that they are more robust to the disturbance in data distribution that happens when applied to another scenario or a scenario evolves over time (like new users, new items, and new interactions occur in recommender systems). Besides, as for machine learning-based models, different hyperparameter settings would lead to fluctuated performance, some of which could be overshadowed by the AIProbS even if its average performance was better, as shown in the following experimental results.

**Figure 1:**
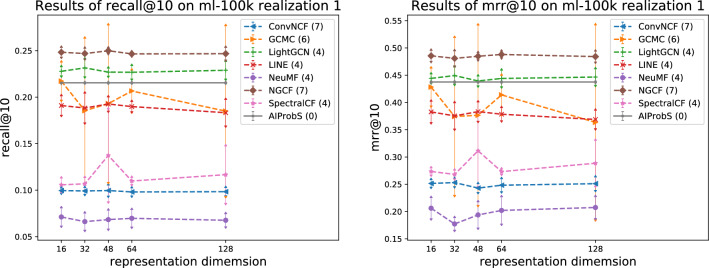
Relation between model precision and representation dimension reflected on ml-100k realization 1.

**Figure 2:**
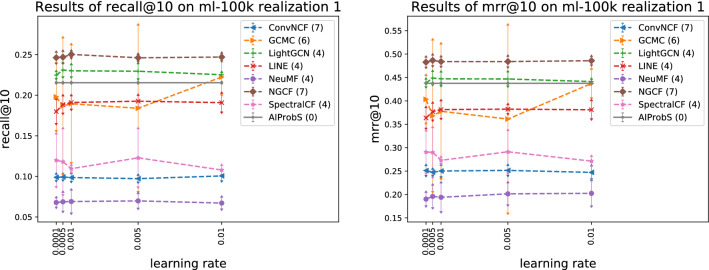
Relation between model precision and learning rate reflected on ml-100k realization 1.

**Figure 3:**
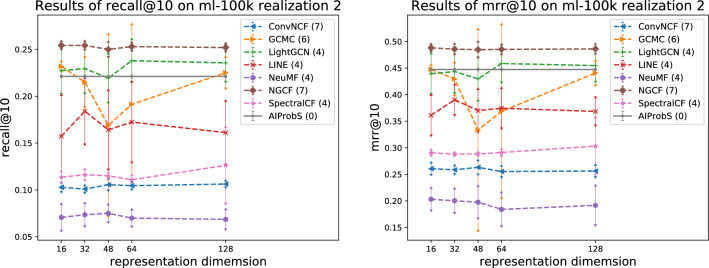
Relation between model precision and representation dimension reflected on ml-100k realization 2.

**Figure 4:**
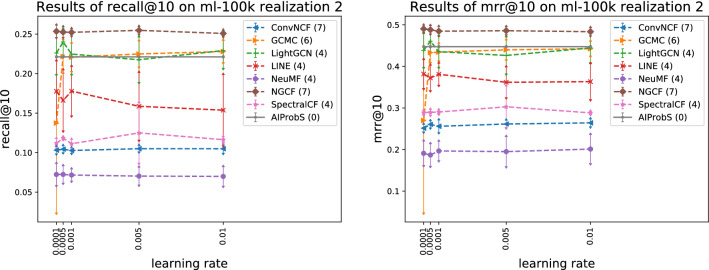
Relation between model precision and learning rate reflected on ml-100k realization 2.

On two realizations of MovieLens 100k for instance, Figs. 1, 2, 3, and 4 present the relations between the different settings of two hyperparameters (i.e., vector dimension and learning rating) and a model’s average precision when one hyperparameter is fixed and others are set with common-used values within a specified search range, where the standard deviation of precision is presented by error arrow at a data point and each model’s number of hyperparameters is presented in parentheses, reflecting a model’s magnitude of performance fluctuation associated with setting disturbance, which actually can quantify the model’s robustness. As shown in Fig. 1, the AIProbS had a stable performance on recall@10, since the dimension of the learned representation vectors by it is determined. However, as a hyperparameter different settings of the vector dimension can largely affect machine learning-based baselines’ prediction precision. Although the performance on recall@10 of ConvNCF, LightGCN, and NGCF of different representation dimensions was relatively stable among machine learning-based baselines, that of GCMC and SpectralCF largely fluctuated with the change of the vector dimension. For example, as for SpectralCF when the vector dimension is set to 48 its average precision could be around $27\%$ higher than that when being set to 16. On top of that, even when the vector dimension of SpectralCF is set to 48, seemingly the optimal choice, its performance on recall@10 still faces a $125\%$ gap between the peaks of performance, flowing from the different settings of other hyperparameters when the vector dimension fixed. Similar fluctuations in machine learning-based baselines’ precision were revealed by the performance under different settings of the vector dimension on mrr@10 and by results shown in Fig. 2 when considering the learning rate as the controlled hyperparameter. Attributed to such the indeterminable performance of machine learning methods, one may have to repeatedly try different hyperparameter settings for a machine learning-based model to search out the optimal (or sub-optimal) one, which is definitely computing resources-consuming and time costly. If an insufficient searching process turns out improper hyperparameter settings, the model could even end up with its worst performance. In contrast, only one implementation is enough for the AIProbS to reach its optimal performance, without any tuning process.

It may be said, though, that determining a machine learning-based model’s optimal (or sub-optimal) hyperparameter settings is burdensome but can be once and for all built on one data set. However, it is not a fact. Similar experiments were done on another realization of MovieLens 100k and the results presented in Figs. 3 and 4 revealed that the optimal hyperparameter settings of a machine learning-based model would be changed with the change of data set. For example, as shown in Fig.1 the optimal vector dimension of SpectralCF was 48 on MovieLens 100k realization 1 but that on MovieLens 100k realization 2 was changed to 128, as shown in Fig. 3. As a result, the tuning process for searching out the optimal hyperparameter settings of a machine learning-based model on another data set appears to be inevitable. In contrast, with the change of data set the vector dimension of the AIProbS is still automatically determined, once implemented.

### Interpretability analysis

Built on the instance in Fig. S1 in Appendix D, this section intuitively explains why the AIProbS model still achieved higher precision than those of machine learning-based models on small data sets and discusses their interpretability in this case.

In the first place, machine learning-based methods are used to learn the nodal representations of the seven nodes in Fig. S1 in Appendix D that can be taken as a training set. Since the dimension of nodal representation learned by machine learning-based models is not determined and, as revealed in the previous section, even if it was specified the models will frequently work out different performances, here two realizations correspond to two different specified nodal representation dimensions (i.e., dim = 3 and dim = 4) were run for instance. As presented in Table 5, the nodal representations learned by machine learning methods were just varied numbers without interpretable meanings related to reality because they conformed to no seeming rules. For example, on the two independent realizations, why did a machine learning-based model specified with the same dimension learned different nodal representations whose relative sizes of elements changed a lot? If they had practical meanings, this should not happen, especially with the change of the numbers’ relative sizes, since a mapping from the numbers to their possible practical meanings does not exist. Besides, the dimension of these nodal representations learned by machine learning methods was also not interpreted. If did, why did the learned representations were with different effective dimensions? Which one should be chosen and why? Recognizing that, in practical applications like recommender systems, it would be helpless for personnel to understand the business based on these not interpreted results. For example, why did the similarity between *A* and *a* calculated based on their representations learned built on different realizations vary? Also, one can not understand why machine learning methods’ performance is often fluctuated with respect to different hyperparameters and realizations, as revealed in the previous section, not to mention how to determine the appropriate ones without repeatedly trying out the tuning process, which is computing resource-consuming.

**Table 5:** Nodal representations learned by machine learning methods.

	dim $=$ 3, realization 1	dim $=$ 3, realization 2	dim $=$ 4, realization 1	dim $=$ 4, realization 2
A	$(-0.546, -0.121, -0.657)$	$(-0.200, -0.212, 0.126)$	$(-0.088, -0.544, 0.276, -0.195)$	$(0.411, -0.322, 0.634, 0.112)$
B	$(-0.760, 0.039, -0.146)$	$(-0.009, -0.415, 0.207)$	$(-0.002, -0.265, 0.141, 0.181)$	$(0.233, -0.284, 0.094, -0.346)$
C	$(-0.324, 0.061, -0.277)$	(0.203, 0.127, 0.426)	(0.195, 0.250, 0.414, 0.342)	$(0.137, -0.299, 0.365, -0.117)$
a	$(-0.385, 0.069, -0.310)$	$(-0.095, -0.166, -0.038)$	$(-0.105, -0.384, 0.141, -0.186)$	$(0.198, -0.177, 0.387, 0.134)$
b	$(-0.354, -0.123, -0.575)$	(0.020, 0.243, 0.312)	$(0.063, -0.047, 0.360, 0.039)$	$(0.246, -0.218, 0.512, 0.097)$
c	$(-0.571, -0.162, -0.411)$	$(-0.230, -0.550, 0.139)$	$(-0.067, -0.545, 0.180, -0.031)$	$(0.328, -0.314, 0.275, -0.171)$
d	$(-0.566, 0.135, -0.068)$	$(0.168, -0.116, 0.396)$	(0.113, 0.107, 0.292, 0.328)	$(0.151, -0.291, 0.145, -0.335)$

In contrast, built on the instance in Fig. S1 in Appendix D, the nodal representations generated by the AIProbS were both interpreted in elements and determined in dimension. First, the nodal representations generated by the AIProbS can preserve a node’s multidimensional influence in reality. This can be seen in Fig. S1. For example, the nodal representation of *A* is $\{3,2\}$, the largest one among others, which can preserve that *A* has the highest overall influence since it can be intuitively observed that *A* has the most neighbors in topology. Yet the nodal representation of *a* is $\{1,1\}$, one of the lowest ones among others, representing that *a* has the lowest overall influence since it is only connected to one node. In practice, these interpreted elements can be used to understand the business. For example, if recommender systems, one can analyze more characteristics of the most influential node *A*, whose passions are most easily inoculated to others, to figure out what his neighbors might be interested in. Second, since the dimension and elements of the nodal representations generated by the AIProbS are determined, the calculated similarity between nodes is always not varied, which can be utilized in recommender systems for reliable analysis of user relationships, item categories, and something.

Attributed to these properties, the AIProbS could outperform machine learning-based methods on small data sets, as presented in Table 2. It is long claimed that the distribution of train data is supposed to be the same (or closely similar) to that of test data for machine learning methods to perform well: the huger their data scales, the more possible they share a common distribution, the more power a machine learning method would show up built on them. However, when the scale of data is small like LastFM and MovieLens 100K illustrated in Table 1, it might be not sufficient to establish the distribution relation between training and test data; then the performance of machine learning-based methods could be confined, indicating that small data could be not enough to well fit a machine learning-based model, let alone the precise similarity between nodes. In contrast, the AIProbS is able to make the best use of small data to generate the similarities reliably in this case.

All in all, in the sense that the AIProbS provides a good trade-off with machine learning-based models on precision, interpretability, and determinacy.

## Discussion

This article proposes two determinable and interpretable node representation methods. Different from other attempts like automated machine learning methods by computer scientists or learning theory by mathematicians to search out and analyze the sub-optimal (or optimal) representation dimension of a machine learning-based network representation model and to interpret the implementing process and the results come out of the model, from a perspective of physics the two proposed methods can substantially generate nodal representations with a determined dimension and interpretable elements, reaching its optimal performance once implemented. After utilizing these representations in link prediction for bipartite networks, experimental results showed that the AIProbS can make a good trade-off with machine learning-based models on precision, determinacy, and interpretability, indicating the effectiveness of nodal representations generated by the two proposed representation methods.

Importantly, these methods with good generalization may motivate further research. For example, nodal representations generated by the two proposed methods can also be utilized in machine learning-based models as initial features, and the AIProbS provides a unified architecture that various nodal representations generated by other methods, like machine learning-based methods, can be integrated, which may further improve the precision of link prediction. Besides, although this work only evaluated the performance of the AIProbS in recommender systems, more in other applications of link prediction could be tried in further research.

Nevertheless, like any model under the effect of the no-free-lunch theorem^45^ that no model can always perform well enough as expected in different scenarios, the AIProbS has its disadvantages in some scenarios. Granted that when concentrating on data sets in small and middle scales it achieved a good performance, which in practice can potentially contribute to companies without enough input data or computing resources for machine learning-based methods, with the increase in data scales, the AIProbS overall underperformed machine learning-based models on precision. Although the AIProbS can make a good trade-off with machine learning methods on precision and interpretability, in some applications where results’ interpretability is unnecessary, like computer vision, healthcare, and finance, machine learning methods seem like a better choice. Besides, since quantum machine learning is usually claimed as the next generation of machine learning, which can exponentially uplift a model’s computing efficiency, would costly hyperparameter tuning be no longer an apprehension in the future? In other words, would determinable network representation that could sacrifice some precision but not representation learning-based (i.e., machine learning-based) methods that are adept in precision still be worthy of quantum computing devices in the future?

## Supplementary Information

Supplementary Information.

## Data Availability

Data and codes are available at (https://github.com/pitteryue/AIProbS).
