# Supplementary material for: Determinable and interpretable network representation for link prediction
Source: Sci Rep. 2022 Oct 20;12:17579. doi: 10.1038/s41598-022-21607-4 (PMC9585049; doi:10.1038/s41598-022-21607-4)
Supplement: Supplementary file 1 — Supplementary Information. [file 41598_2022_21607_MOESM1_ESM.pdf]

Supplementary materials for determinable and interpretable network representation for link prediction

Yue Deng[1,*]

[1]Institute of Fundamental and Frontier Sciences, University of Electronic Science and Technology of China, Chengdu 611731, People's Republic of China
[*]201921210214@std.uestc.edu.cn

A Proof of theorem 2.2

A.1 Preliminaries

Before proving theorem 2.2, essential preliminaries are presented as follow.

Definition A.1. *A simple matrix is defined as a matrix $A^{n\times n}$ when the algebraic and geometrical multiplicities of each of its eigenvalues are equal.*

Definition A.2. *A normal matrix is defined as a matrix $A^{n\times n}$ if and only if $AA^T = A^TA$ when $A^{n\times n}$ is a real matrix or $AA^H = A^HA$ when $A^{n\times n}$ is a complex matrix.*

Definition A.3. *An idempotent matrix is defined as a matrix $A^{n\times n}$ if and only if $A^2 = A$.*

Lemma A.1. *Let A be a $n \times n$ simple matrix with eigenvalues $\lambda_1, \lambda_2, ..., \lambda_n$. Then A can be diagonalized by*

$$P^{-1}AP = \mathrm{diag}(\lambda_1, \lambda_2, ..., \lambda_n), \tag{1}$$

where P is an invertible matrix.

Proof. For any matrix A, there exists an invertible matrix P such that $P^{-1}AP = J = \mathrm{diag}(J_1(\lambda_1), J_2(\lambda_2), ..., J_r(\lambda_r))$, where

$$J_i(\lambda_i) = \begin{pmatrix} \lambda_i & 1 & & \\ & \cdots & \cdots & \\ & & \lambda_i & 1 \\ & & & \lambda_i \end{pmatrix} \tag{2}$$

is called a Jordan block, and the matrix J is called the Jordan standard form of A.

The number of Jordan blocks corresponding to the same eigenvalue is the geometric multiplicity of that eigenvalue. The sum of the orders of all Jordan blocks corresponding to the same eigenvalue is the algebraic multiplicity of that eigenvalue

By the definition of a simple matrix, where the algebraic and geometrical multiplicities of each of its eigenvalues are equal, the Jordan blocks of a simple matrix $A^{n\times n}$ can be denoted by $J_i(\lambda_i) = (\lambda_i)$, a 1×1 matrix. Then, for a simple matrix $A^{n\times n}$, we have:

$$P^{-1}AP = \begin{pmatrix} J_i(\lambda_1) & & & \\ & J_2(\lambda_2) & & \\ & & \cdots & \\ & & & J_n(\lambda_n) \end{pmatrix} = \begin{pmatrix} \lambda_1 & & & \\ & \lambda_2 & & \\ & & \cdots & \\ & & & \lambda_n \end{pmatrix}. \tag{3}$$

□

A.2 Proof of Theorem 2.2

Apparently, as a symmetric matrix, the adjacency matrix $B^{(m+n)\times(m+n)}$ is a normal matrix, so it is a simple matrix. Then, by Lemma A.1, we have:

$$B = P\mathrm{diag}(\lambda_1, \lambda_2, ..., \lambda_{m+n})P^{-1}, \tag{4}$$

where $\lambda_1, \lambda_2, ..., \lambda_{m+n}$ are the $m+n$ eigenvalues of B.

Given P is an invertible matrix, we can denote

$$P = (v_1, v_2, ..., v_{m+n}) \tag{5}$$

consisting of linearly independent column vectors v_i, where $Bv_i = \lambda_i v_i (i = 1, 2, ..., m+n)$. Since $BP = P\text{diag}(\lambda_1, \lambda_2, ..., \lambda_{m+n})$, we have $B = P\text{diag}(\lambda_1, \lambda_2, ..., \lambda_{m+n})P^{-1}$. Then:

$$B^T = (P^{-1})^T \text{diag}(\lambda_1, \lambda_2, ..., \lambda_{m+n})P^T \tag{6}$$

$$= (P^T)^{-1} \text{diag}(\lambda_1, \lambda_2, ..., \lambda_{m+n})P^T. \tag{7}$$

Let $\omega_1, \omega_2, ..., \omega_{m+n}$ be the $m+n$ eigenvectors of B^T, which are linearly independent column vectors, we have $B^T(\omega_1, \omega_2, ..., \omega_{m+n}) = (\omega_1, \omega_2, ..., \omega_{m+n})\text{diag}(\lambda_1, \lambda_2, ..., \lambda_{m+n})$. Then:

$$B^T = (\omega_1, \omega_2, ..., \omega_{m+n})\text{diag}(\lambda_1, \lambda_2, ..., \lambda_{m+n})(\omega_1, \omega_2, ..., \omega_{m+n})^{-1}. \tag{8}$$

Apparently, combining Eq. (6) and Eq. (8) we get $(P^T)^{-1} = (\omega_1, \omega_2, ..., \omega_{m+n})$, so we can denote $P^{-1} = ((P^{-1})^T)^T = (((P^T)^{-1})^T = (\omega_1, \omega_2, ..., \omega_{m+n})^T$. That is:

$$P^{-1} = \begin{pmatrix} \omega_1^T \\ \omega_2^T \\ ... \\ \omega_{m+n}^T \end{pmatrix}. \tag{9}$$

Based on Eqs. (4), (5) and (9), we can decompose B by

$$B = \begin{pmatrix} v_1, v_2, ..., v_{m+n} \end{pmatrix} \begin{pmatrix} \lambda_1 & 0 & ... & 0 \\ 0 & \lambda_2 & ... & 0 \\ ... & ... & ... & ... \\ 0 & 0 & ... & \lambda_{m+n} \end{pmatrix} \begin{pmatrix} \omega_1^T \\ \omega_2^T \\ ... \\ \omega_{m+n}^T \end{pmatrix} \tag{10}$$

$$= \textstyle\sum_{i=1}^{m+n} \lambda_i v_i \omega_i^T. \tag{11}$$

Furthermore, let $B_i = v_i\omega_i^T$. Since $P^{-1}P = E_{m+n}$ (*i.e.*, $\omega_i^T v_j = \begin{cases} 1 & j = i, \\ 0 & j \neq i, \end{cases}$), $B_i(i = 1, 2, ..., m+n)$ are idempotent matrices satisfying $B_iB_j = \begin{cases} B_i & j = i, \\ 0 & j \neq i, \end{cases}$.

Finally, we come to the theorem that B can be decomposed by

$$B = \sum_{i=1}^{m+n} \lambda_i B_i, \tag{12}$$

where λ_i is the i-th eigenvalue of $B^{(m+n)\times(m+n)}$ and B_i is the corresponding idempotent matrix. □

B Proof of theorem 2.3

Proof. Since $R = A \cdot (D_I \circ A)^T \cdot (D_U \circ A) \Leftrightarrow R^T = (D_U \circ A)^T \cdot (D_I \circ A) \cdot A^T$ holds, here let the operator $T' = (D_U \circ A)^T \cdot (D_I \circ A)$.

In the first place, construct a non-empty complete metric space $(\mathbb{R}^{n\times m}, d_{\max})$ based on $\mathbb{R}^{n\times m}$. Since any two norms on a finite-dimensional linear space are equivalent, for simplicity we choose the norm-induced metric $d_{\max}(A,B) = \max\limits_{i,j}\{|a_{ij}|\}, 1 \leq i \leq m, 1 \leq j \leq n$. It is easy to prove that $(\mathbb{R}^{n\times m}, d_{\max})$ is a non-empty complete metric space, as follows.

There is no doubt that $(\mathbb{R}^{n\times m}, d_{\max})$ is a metric space. Furthermore, assume $\{X\}$ is a Cauchy sequence in $\mathbb{R}^{n\times m}$, we have that $\forall \varepsilon > 0, \exists N \in \mathbb{N}$, s.t., $m, n > N, d(X^{(n)} - X^{(m)}) = \max_{i,j}\{|X_{ij}^{(n)} - X_{ij}^{(m)}|\} < \varepsilon$. For any sequence $\{X_{ij}\}$ with fixed (i, j), we have that $\forall \varepsilon > 0, \exists N \in \mathbb{N}$, s.t., $m, n > N, d(X_{ij}^{(n)}, X_{ij}^{(m)}) \leq \max_{i,j}\{|X_{ij}^{(n)} - X_{ij}^{(m)}|\} < \varepsilon$. So sequence $\{X_{ij}\}$ is a Cauchy sequence. Since $\mathbb{R}$ is complete, X_{ij}^* exists such that $X_{ij}^{(n)} \longrightarrow X_{ij}^*$ when $n \longrightarrow \infty$, where $X_{ij}^* \in \mathbb{R}$. So X^* exists such that $X^{(n)} \longrightarrow X^*$, when $n \longrightarrow \infty$. Apparently, $X^* \in \mathbb{R}^{n\times m}$. Therefore, $(\mathbb{R}^{n\times m}, d_{\max})$ is a non-empty complete metric space.

Then, according to the Banach fixed point theorem on a non-empty complete metric space, the operator

$$T' = (D_U \circ A)^T \cdot (D_I \circ A) = \begin{pmatrix} \frac{1}{K_{I_1}} \sum_{h=1}^{m} \frac{a_{h1}a_{h1}}{K_{U_h}} & \frac{1}{K_{I_2}} \sum_{h=1}^{m} \frac{a_{h1}a_{h2}}{K_{U_h}} & \cdots & \frac{1}{K_{I_n}} \sum_{h=1}^{m} \frac{a_{h1}a_{hn}}{K_{U_h}} \\ \frac{1}{K_{I_1}} \sum_{h=1}^{m} \frac{a_{h2}a_{h1}}{K_{U_h}} & \frac{1}{K_{I_2}} \sum_{h=1}^{m} \frac{a_{h2}a_{h2}}{K_{U_h}} & \cdots & \frac{1}{K_{I_n}} \sum_{h=1}^{m} \frac{a_{h2}a_{hn}}{K_{U_h}} \\ \cdots & \cdots & \cdots & \cdots \\ \frac{1}{K_{I_1}} \sum_{h=1}^{m} \frac{a_{hn}a_{h1}}{K_{U_h}} & \frac{1}{K_{I_2}} \sum_{h=1}^{m} \frac{a_{hn}a_{h2}}{K_{U_h}} & \cdots & \frac{1}{K_{I_n}} \sum_{h=1}^{m} \frac{a_{hn}a_{hn}}{K_{U_h}} \end{pmatrix}, \tag{13}$$

where $a_{**} = 0$ or 1 and $\frac{a_{**}a_{**}}{K_*} = 0$ if $K_* = 0$, is a contraction mapping on $(\mathbb{R}^{n\times m}, d_{\max})$. Finally, we have that T is a contraction mapping on $(\mathbb{R}^{m\times n}, d_{\max})$, meaning that the iterative process $A \longleftarrow A \cdot T$ is convergent to a fixed point A^*. □

C Other combinations of the AIProbS

The user-item similarity based on F_U and F_I can be calculated by other metrics, like covariance (Cov), dot product, Euclidean Distance (ED) and Pearson correlation coefficient (Pearson), as follows.

Combinations	Recall@10	MRR@10	NDCG@10
**cosine + M-M + P (used)**	**0.215**	**0.434**	**0.248**
cosine + M-M	0.167	0.405	0.217
cosine	0.167	0.405	0.217
Cov + M-M + P	0.132	0.318	0.166
Cov + M-M	0.125	0.303	0.158
Cov	0.125	0.303	0.158
dot_product + M-M + P	0.180	0.398	0.218
dot_product + M-M	0.136	0.343	0.180
dot_product	0.136	0.343	0.180
ED + M-M + P	0.197	0.411	0.231
ED + M-M	0.147	0.366	0.193
ED	0.147	0.366	0.193
Pearson + M-M + P	0.207	0.419	0.238
Pearson + M-M	0.158	0.387	0.206
Pearson	0.158	0.387	0.206

Table S1. Results of model precision based on other combinations on MovieLens 100K, as an instance.

C.1 Covariance (Cov)

$$S_{\text{Cov}} = \frac{1}{s}(F_U - \overline{F_U}) \cdot (F_I - \overline{F_I})^T, \tag{14}$$

where $\overline{F_U} = (\alpha^T, \alpha^T, ..., \alpha^T)$, $\overline{F_I} = (\beta^T, \beta^T, ..., \beta^T)$, $\alpha = (\frac{1}{s}\sum_{j=1}^{s} F_{U\,1j}, \frac{1}{s}\sum_{j=1}^{s} F_{U\,2j}, ...,$ $\frac{1}{s}\sum_{j=1}^{s} F_{U\,mj})$, $\beta = (\frac{1}{s}\sum_{j=1}^{s} F_{I\,1j}, \frac{1}{s}\sum_{j=1}^{s} F_{I\,2j}, ..., \frac{1}{s}\sum_{j=1}^{s} F_{I\,nj})$.

C.2 dot product

$$S_{\text{dot\_product}} = F_U \cdot F_I. \tag{15}$$

C.3 Euclidean distance (ED)

$$S_{\text{ED}} = S = \sqrt{|-2F_U \cdot F_I^T + \mathscr{F_U} + \mathscr{F_I}|}, \tag{16}$$

where $\mathscr{F_U}^{m\times n} = (\alpha^T, \alpha^T, ..., \alpha^T)$, $\mathscr{F_I}^{m\times n} = (\beta, \beta, ..., \beta)^T$, $\alpha = (\sqrt{\sum_{j=1}^{s} F_{U\,1j}^2}, \sqrt{\sum_{j=1}^{s} F_{U\,2j}^2}, ...,$ $\sqrt{\sum_{j=1}^{s} F_{U\,mj}^2})$, $\beta = (\sqrt{\sum_{j=1}^{s} F_{I\,1j}^2}, \sqrt{\sum_{j=1}^{s} F_{I\,2j}^2}, ..., \sqrt{\sum_{j=1}^{s} F_{I\,nj}^2})$.

C.4 Pearson correlation coefficient (Pearson)

$$s_{\text{Pearson}} = \frac{(F_U - \overline{F_U})(F_I - \overline{F_I})}{\alpha^T \beta}, \tag{17}$$

where $\alpha = \left(\sqrt{\sum_{j=1}^{s}(F_{U1j} - \overline{F_{U1*}})^2}, \sqrt{\sum_{j=1}^{s}(F_{U2j} - \overline{F_{U2*}})^2}, ..., \sqrt{\sum_{j=1}^{s}(F_{Umj} - \overline{F_{Um*}})^2}\right)$,

$\beta = \left(\sqrt{\sum_{j=1}^{s}(F_{I1j} - \overline{F_{I1*}})^2}, \sqrt{\sum_{j=1}^{s}(F_{I2j} - \overline{F_{I2*}})^2}, ..., \sqrt{\sum_{j=1}^{s}(F_{Inj} - \overline{F_{In*}})^2}\right)$.

After combining these metrics with the max-min normalization (M-M) operation by Eq. (3) or the proportioning (P) operation by Eq. (4), the AIProbS model of diverse versions can be constructed. Tab. S1 presents the results on model precision of these new combinations, based on the evaluation settings designed in this article and MovieLens 100K for an instance, among which the combination that *cosine* + *M-M* + *P* used in this article achieves the best performance.

The reason why these similarity metrics were chosen for extended experiments lies in two main points. First, these metrics are normal and basic ones. Although more advanced and state-of-the-art similarity metrics could be taken as alternatives for other combinations, here only the common-used ones were evaluated. Second, granted that some metrics like Jaccard and Adamic-Adar are normal ones, they can not be directly written as a matrix formation to directly support the parallel computing for Python, which also require a search and traversal operation to count, say, the intersection and union of sets that would abate the efficiency of the AIProbS.

D The pseudocodes and schematics of AIProbS

The pseudocodes of the AIProbS are displayed as follows.

Pseudocodes. The AIProbS model

Input: implicit interactions between m users and n items,
the length N of the recommendation list.
Output: user's top-N recommendations.
1 : Construct Adjacency Matrix: construct $A^{m \times n}$ of the input;
2 : Generate Feature: generate F_U, F_I by methods in this article;
3 : Calculate Similarity: calculate $S^{m \times n}$ by Eq. (2);
4 : foreach *row vector* $(S \circ A)_{i*}$ *in* $(S \circ A)^{m \times n}$ **do**
5 : max = max($(S \circ A)_{i*}$), min = min($(S \circ A)_{i*}$);
6 : $(S \circ A)_{ij} \leftarrow \frac{(S \circ A)_{ij} - \min}{\max - \min}$, $j = 1, 2, ..., n$;
7 : $W_{U_{ij}} = \frac{1}{(S \circ A)_{ij}} \sum_{k=1}^{n} (S \circ A)_{ik}$, $j = 1, 2, ..., n$;
8 : foreach *column vector* $(S \circ A)_{*j}$ *in* $(S \circ A)^{m \times n}$ **do**
9 : max = max($(S \circ A)_{*j}$), min = min($(S \circ A)_{*j}$);
10: $(S \circ A)_{ij} \leftarrow \frac{(S \circ A)_{ij} - \min}{\max - \min}$, $i = 1, 2, ..., m$;
11: $W_{I_{ij}} = \frac{1}{(S \circ A)_{ij}} \sum_{k=1}^{m} (S \circ A)_{kj}$, $i = 1, 2, ..., m$;
12: $R = A \cdot W_I^T \cdot W_U$;
13: foreach *row vector* R_{i*} *in* R **do**
14: record the column indices of R_{i*}'s top-N elements into L_i;
15: return $L^{m \times N}$ as user's top-N recommendations;

Built on Fig. S1, examples are presented to illustrate the k-core decomposition, the ProbS framework, and the two proposed nodal representation methods in this article as well as their application in link prediction (*i.e.*, the AIProbS model). As shown in Fig. S1, there are seven nodes of two types in the bipartite network, that is, nodes A, B, and C of type one and nodes a, b, c, and d of type two. In recommender systems, the two types of nodes correspond to users and items. Based on the bipartite network, the resource diffusion process of the ProbS framework is presented. For instance, in the first step (from the left nodes to the right nodes), nodes c and d are allocated with 1 unit, respectively, because they are connected with node B. Otherwise, nodes a and b are allocated with 0 unit, respectively. In the second step (from the right nodes to the left nodes), each node distributes

their resources equally and diffuses them to the left nodes. For example, since it is connected to two nodes (nodes A and B), node c distributes its resources equally to $1/2$ and $1/2$ and then diffuses them to nodes A and B. After the second step, the right nodes are allocated accumulated resources diffused from their connected left nodes. For example, the accumulated resources diffused from nodes c and d to node B is $1/2+1/2=1$. In the third step (from the left nodes to the right nodes), the same diffusion process is completed. Finally, the right nodes are allocated accumulated resources diffused from their connected right nodes, which can be used to measure their similarity with node B, respectively. For example, since node b is allocated with more accumulated resources than node a, it can be predicted that node b is more likely to be connected with node B. However, due to the mechanism of the ProbS framework that equally distributes resources without distinguishing structural characteristics of different networks, underlying patterns in the network that might be utilized to measure the similarity between nodes are still hidden. In fact, the more precise the similarity between nodes is measured, the better performance the link prediction will reach. To make up for the flaw, this work first proposes two nodal representation methods.

As for method one, operator $\mathscr{H}$ of the DHC-E theorem can be used to generate the H-indices sequences for each node in the bipartite network. For instance, in the beginning, the degree of node A is calculated as 3, which is taken as the zero-order H-index $h_A^{(0)}$ of node A. At the same time, the zero-order H-index of the nodes connected with node A can be calculated as $h_a^{(0)}=1, h_b^{(0)}=2$, and $h_c^{(0)}=2$, respectively. Since there are at least 2 nodes (*i.e.*, nodes b and c) with a degree no less than 2, the first-order H-index $h_A^{(1)}$ of node A is 2. In the same way, it has $h_A^{(2)}=2$. When going to the third step, the H-indices of all nodes in the network are the same as in the previous step, and the whole process converges. Finally, the H-indices sequences of all nodes are obtained. For example, node A's H-indices sequence is $\{3,2\}$. These sequences are taken as nodal representations. As for method two, after generating the idempotent matrices corresponding to each node, the same process as method one is conducted, obtaining the H-indices sequences of all nodes in the network.

After obtaining all nodes' nodal representations, the similarity between node pairs can be calculated, which is used to control the distribution of resources in the ProbS framework by appointing edge weights. For instance, since the H-indices sequences of nodes A, a, b, and c generated, say, by method one are $\{3,2\}$, $\{1,1\}$, $\{2,2\}$, and $\{2,2\}$, respectively, according to the AIProbS model the similarities between them can be calculated as $S_{Aa}=5/\sqrt{26}$, $S_{Ab}=5/\sqrt{26}$, and $S_{Ac}=5/\sqrt{26}$. Then the weights of edges w_{Aa}, w_{Ab}, and w_{Ac} are $1/3$, $1/3$, and $1/3$ respectively, which are used to control the distribution of resources in the diffusion process.

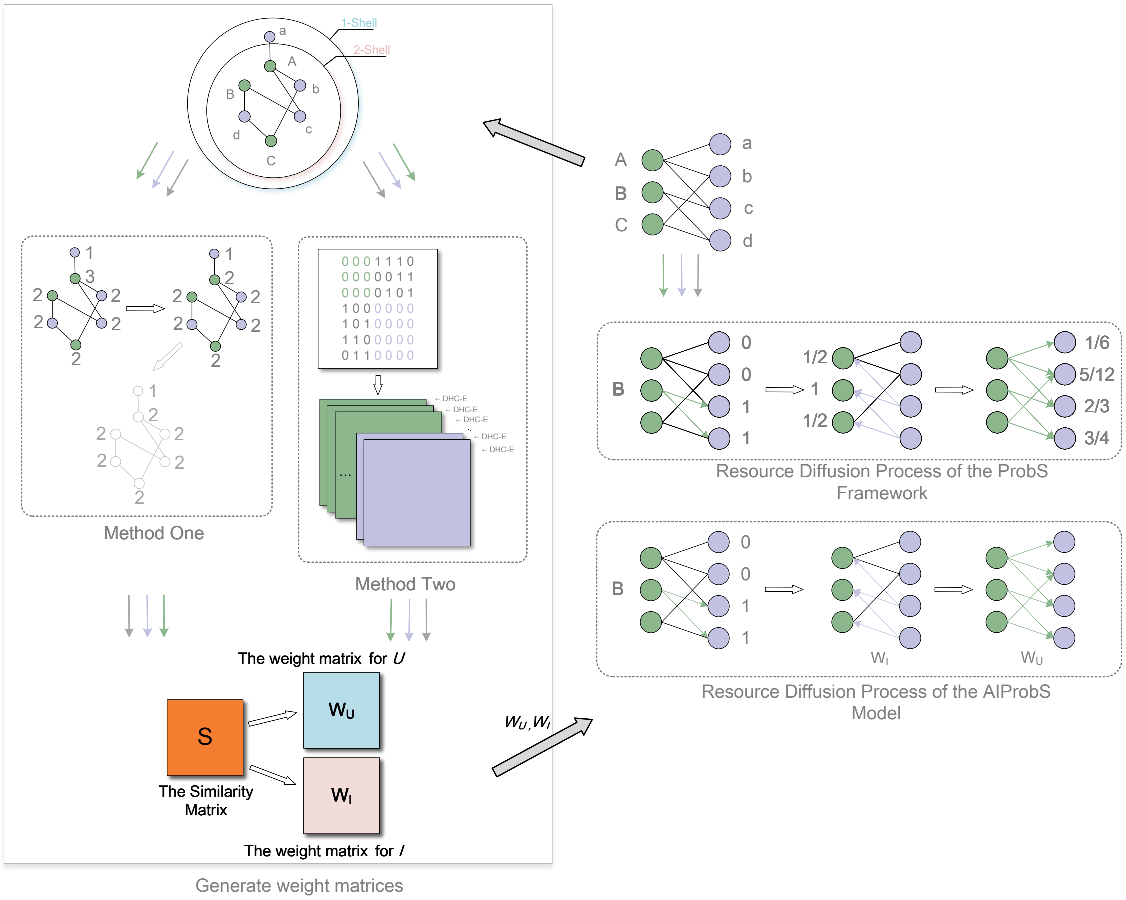

Figure S1. Schematics of the AIProbS model. With a toy example, the schematics illuminate the processes of the two nodal feature generation methods and the recommendation based on the ProbS framework and the AIProbS model for Bob in a movie recommender system.
